# PhylOPDb: a 16S rRNA oligonucleotide probe database for prokaryotic identification

**DOI:** 10.1093/database/bau036

**Published:** 2014-04-26

**Authors:** Faouzi Jaziri, Nicolas Parisot, Anis Abid, Jérémie Denonfoux, Céline Ribière, Cyrielle Gasc, Delphine Boucher, Jean-François Brugère, Antoine Mahul, David R.C. Hill, Eric Peyretaillade, Pierre Peyret

**Affiliations:** ^1^Clermont Université, Université d’Auvergne, EA 4678 CIDAM, BP 10448, F-63001 Clermont-Ferrand, France, ^2^UMR CNRS 6158, ISIMA/LIMOS, Clermont Université, Université Blaise Pascal, F-63173 Aubière, France, ^3^CNRS, UMR 6023, LMGE, F-63171 Aubière, France and ^4^Clermont Université, CRRI, F-63177 Aubière, France

## Abstract

In recent years, high-throughput molecular tools have led to an exponential growth of available 16S rRNA gene sequences. Incorporating such data, molecular tools based on target-probe hybridization were developed to monitor microbial communities within complex environments. Unfortunately, only a few 16S rRNA gene-targeted probe collections were described. Here, we present PhylOPDb, an online resource for a comprehensive phylogenetic oligonucleotide probe database. PhylOPDb provides a convivial and easy-to-use web interface to browse both regular and explorative 16S rRNA-targeted probes. Such probes set or subset could be used to globally monitor known and unknown prokaryotic communities through various techniques including DNA microarrays, polymerase chain reaction (PCR), fluorescent *in situ* hybridization (FISH), targeted gene capture or *in silico* rapid sequence identification. PhylOPDb contains 74 003 25-mer probes targeting 2178 genera including *Bacteria* and *Archaea*.

**Database URL:** http://g2im.u-clermont1.fr/phylopdb/

## Background

Prokaryotes are the most important and diverse group of organisms, widely distributed across almost all environmental habitats, even the most extreme, and involved in various ecological and environmental processes. However, because of this tremendous diversity and the technological limits such as our inability to culture the vast majority of microorganisms (1), our current vision of the microbial world is still incomplete. Thus, the comprehension of prokaryote diversity, abundance and dynamics remains a major challenge of microbial ecology. To overcome the limitations of the culture-based methods, some molecular tools were therefore developed to survey prokaryotic communities (2) such as polymerase chain reaction (PCR)-based DNA fingerprints, fluorescent *in situ* hybridization (FISH) or clone libraries sequencing. Over the past decades, most promising high-throughput approaches were developed including DNA microarrays and next-generation sequencing that can also be coupled to gene capture (3).

Targeting the small subunit (SSU) ribosomal RNA gene, i.e. 16S rRNA gene, is particularly well adapted to survey prokaryotic communities in complex environments, as it contains highly conserved and variable moieties permitting reliable and detailed bacterial classification. Moreover, the advent of many PCR-based approaches, as well as sequencing projects, has led to the explosion of 16S rRNA gene sequences now available in major specialized sequence repositories, such as Greengenes (4), SILVA (5) and RDP (6). Taking into account this amount of data, high-throughput tools using the SSU rRNA biomarker such as phylogenetic oligonucleotide arrays (POAs) have been developed. Several tools were therefore proposed to select phylogenetic probes such as PRIMROSE (7), ARB PROBE_DESIGN (8), ORMA (9) or CaSSiS (10, 11). Unfortunately, most of these programs are not well-suited for large-scale probe design. Designing probes for a large group of sequences requires considerable computational resources and can take up to few days for only one design. Only CaSSiS was implemented for large-scale sequence data sets. Furthermore, all of these tools allow selecting probes targeting only known microbial communities with available sequences in public environmental databases. However, it is also important to define explorative probes that can detect uncharacterized phylogenetic signatures and anticipate genetic variations (12). Currently, only four software programs allow the selection of explorative phylogenetic probes: PhylArray (13), PhylGrid (14), KASpOD (15) and MetaExploArrays (16).

Although numerous oligonucleotide probes have been reported, available collections of rRNA-targeted oligonucleotide probes are rare. The oligonucleotide probe database (OPD) (17) was proposed in 1996 to collect tested phylogenetic oligonucleotide probes. The last data set of OPD listed 96 primers and probes targeting small and large subunit rRNA. However, OPD has not been updated since 1997 and is no longer available online. More recently, ‘probeBase’ (18), an online resource for published rRNA-targeted oligonucleotide probes and associated information, was established in 2002. It currently includes 2788 probes (status January 2014).

Here, we present PhylOPDb (‘phylogenetic oligonucleotide probe database’), a comprehensive phylogenetic OPD targeting 16S rRNA gene sequences. We used two high-throughput probe design software, PhylGrid and KASpOD, to select both regular and explorative 16S rRNA gene-targeted oligonucleotide probes. PhylOPDb is composed of 74 003 probes of 25 mer targeting 2178 genera including *Bacteria* and *Archaea*.

## Database construction and development

### Probe design using PhylGrid

**Figure 1.**
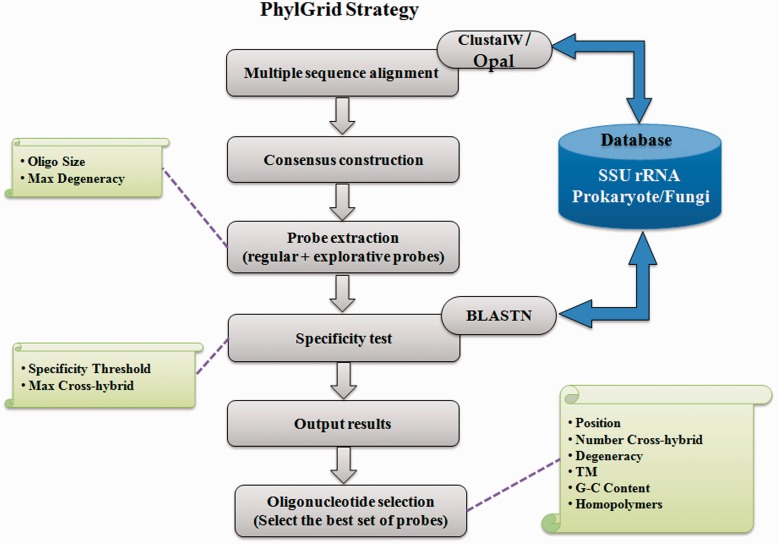
Overview of the PhylGrid algorithm.

PhylGrid (14) is a large-scale probe design software linked to the EGI grid. It is an improvement of the PhylArray algorithm presented in Militon *et al.* (13) that allows defining regular and explorative oligonucleotide probes targeting SSU rRNA genes at any phylogenetic level (Figure 1).

The PhylGrid probe design was based on a custom 16S rDNA-curated sequence database originating from the EMBL. All SSU rDNA sequences downloaded from the prokaryotic (PRO) and environmental (ENV) divisions of the EMBL nucleotide sequence database were used as a reference to build this database. First, 16S rDNA gene sequences were extracted and filtered according to their quality and size. Only sequences that met the following criteria were kept: (i) sequence length is between 1200 and 1600 nucleotides, (ii) sequence is assigned to the genus level in EMBL database, (iii) the percentage of ambiguous nucleotides is <1% and (iv) the maximum number of consecutive unknown bases must not exceed five. Then, extracted sequences were grouped at the genus taxonomic rank according to the NCBI taxonomy database. For each genus, sequence orientation of all sequences was checked using BLASTN (19) and redundancy was eliminated using BLASTCLUST (19). Finally, a K-means approach was implemented to check for badly annotated sequences that may prevent selecting specific probes for this group. Eventually, 66 075 rDNA (16S) gene sequences representing 2069 prokaryotic genera were obtained.

Using this custom 16S rRNA gene sequences database, a total of 3 553 975 degenerate probes of 25 mer were selected. The probe length of 25 nucleotides offers the balance of highest sensitivity and specificity in the presence of a complex background (20, 13). Coverage and specificity tests were performed against the input database using a BLASTN (19) allowing up to two mismatches between a probe and its target. Putative cross-hybridizations were therefore defined when a non-targeted sequence harbours at most two mismatches with a probe. The number of mismatches allowed was chosen taking into the destabilization effect on the probe–target complex and the loss of signal (21). A stringent threshold of two mismatches was set to limit putative cross-hybridizations. It should be noticed that ambiguous nucleotides are considered as mismatches by BLASTN.

Five non-overlapping probes showing the best specificity and coverage were then selected for each genus. During this step, some cross-hybridizing probes may have been selected. Nevertheless, the program ensured that the simultaneous analysis of the five selected probes could not cause misleading interpretations of hybridization data. A set of 19 874 25-mer probes corresponding to 10 320 degenerate probes was obtained.

### Probe design using KASpOD

**Figure 2.**
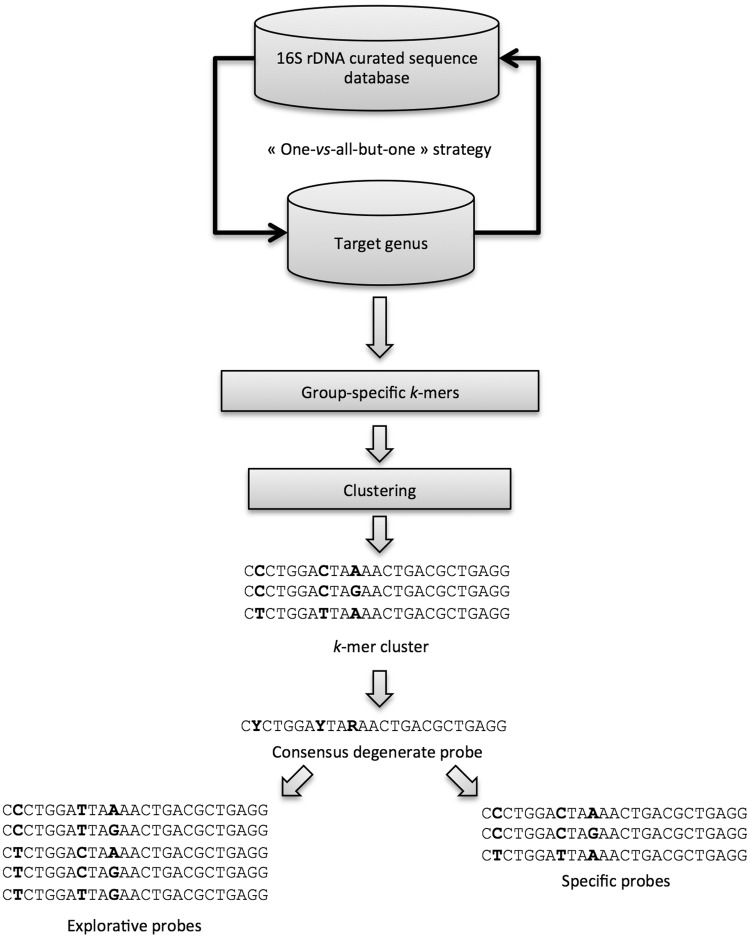
Schematic representation of the KASpOD program workflow.

KASpOD (15) is a fast *k*-mer–based software dedicated to the design of group-covering oligonucleotide probes. It allows selecting highly specific and explorative probes based on large data sets (Figure 2).

The KASpOD probe design was based on the Greengenes database (22). The May 2011 release containing 406 997 sequences was downloaded and extracted from Greengenes. Then, using a custom PERL script, only the sequences assigned to a genus were retained for further analyses. These 310 575 sequences were then sorted by genus into different FASTA files. For each genus, a clustering step was performed at a 100% identity threshold using CD-HIT (23) to remove any redundancies. Moreover, only high-quality sequences were retained: (i) sequence length >1200 nucleotides and (ii) <1% of ambiguous nucleotides. After this processing pipeline, the 16S rDNA database contained 252 250 high-quality sequences. The clustering of the whole database at high-identity thresholds (99, 98 and 97%) coupled with manual curation, allowed removing of potentially badly assigned sequences. Furthermore, some microbial genera were clustered together, as they were hardly distinguishable on the basis of their sequences. Eventually, 252 183 16S rDNA sequences were fed to KASpOD to perform the probe design.

A total of 3 242 105 degenerate candidate probes of 25 mer were designed for 1295 prokaryotic genera. The maximum number of mismatches between a probe and its target was set to two mismatches, and any ambiguous character will be counted as a match if the aligning base is one of the nucleotides represented by the ambiguity code.

The minimal probeset harbouring the best coverage and specificity was then defined. First, the non-overlapping probes were selected within the probes showing no cross-hybridizations. Subsequently, while there were some 16S rDNA sequences, which were not covered by at least three probes, the program selected additional probes with increasing numbers of cross-hybridizations. During this step, the program ensured that no more than two probes show significant cross-hybridization with the same non-targeted genus, thereby avoiding misleading interpretations of hybridization data.

Finally, after the removal of redundant probes with the PhylGrid probes, 54 129 16S rRNA gene-targeted oligonucleotide probes were added to PhylOPDb. 

### PhylOPDb web interface

**Figure 3.**
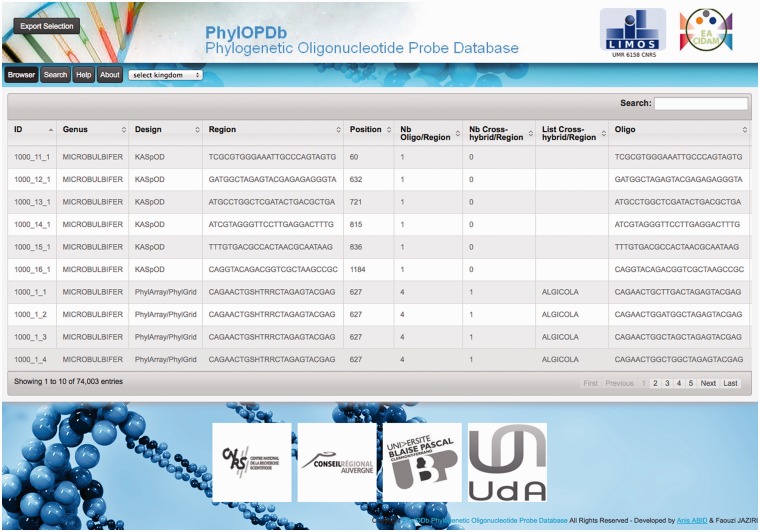
Screenshot of the PhylOPDb web interface.

To make all the phylogenetic oligonucleotide probes easily available, we developed a web interface (Figure 3) to fetch and download the 74 003 probes that compose our oligonucleotide database (PhylOPDb). The website, freely available (http://g2im.u-clermont1.fr/phylopdb/), was implemented using PHP, HTML5, CSS3, JavaScript, jQuery, JSON and MySQL. PhylOPDb will be updated annually adding newly designed probes, removing deprecated probes and re-computing coverage and specificity. Moreover, the 16S rDNA sequences databases used for the probe design can be downloaded through the PhylOPDb web interface.

Based on the EMBL taxonomy, the PhylOPDb web interface provides a hierarchical browse of the database contents. When a taxonomic group is selected, corresponding to a kingdom, phylum, class, order, family or genus, the oligonucleotide probes of this group are then displayed. Selected probes can be downloaded both in CSV and FASTA formats. Furthermore, for each non-degenerate probe, associated information is given: identifier, genus name, sequence, length, corresponding degenerate probe, degeneracy, coverage percentage, position according to the consensus sequence of the genus, putative cross-hybridizations, melting temperature and the probe design tool used.

Probes can also be obtained using a rapid search by keywords that can represent a part of the sequence of a probe, a genus name or a design name (PhylGrid or KASpOD). Only probes that match these keywords are then displayed. Probes can also be fetched through an advanced search using multiple criteria (genus, design, coverage, melting temperature range or number of cross-hybridizations).

## Discussion

Over the past decades, high-throughput molecular tools have opened an unprecedented opportunity for microbiology by enabling the culture-independent genetic study of complex microbial communities, which were so far largely unknown. Among these tools, environmental microarrays, including POAs, are key technologies that are well adapted to profiling environmental communities (21, 24–29). For instance, the PhyloChip (28) is currently the most widely used high-density POA. Nevertheless, 16S rRNA gene-targeted probesets were poorly described and updated. Therefore, PhylOPDb is the most comprehensive SSU rRNA oligonucleotide database by overcoming the currently existing 16S rRNA gene-targeted probe collections. For instance, the entire probeset provided through the PhylOPDb web interface could be used to build a comprehensive POA allowing monitoring of >2000 microbial genera in one experiment. PhylOPDb provides a free and convivial web interface to browse and download a complete 16S rRNA gene-targeted oligonucleotide database composed of 74 003 regular and explorative 25-mers probes covering 2178 prokaryotic genera.

PhylOPDb is also well adapted for other molecular tools using primers or probes (PCR, quantitative PCR, FISH and gene capture) with the availability of group-specific signatures. One of the goals of our database is to exhaustively provide the most specific probes or primers at a fine phylogenetic level. When biologists are interested in specific microbial taxa, it is difficult to reveal them using ‘universal’ probes or primers. For such biological applications, we consider that our database will be helpful. Furthermore, thermodynamics of nucleic acids hybridizations is not fully understood, molecular approaches such as FISH, PCR, DNA microarrays and gene capture are still empiric and need biological validations. With our complete database, biologists could test various probes or primers to select the most adapted to answer their biological questions. To reduce the complexity of probes and primers selection, we indicate in the database degenerate signatures from which specific probes and primers are deduced. The problem of probes accessibility is particularly relevant for the FISH approach. However, it is possible to resolve this difficulty using helper oligonucleotides, as previously described (30). Recently, a promising strategy for reducing the biocomplexity of environmental samples by enriching the desired genomic target using probes before massive sequencing is being adapted for microbial ecology (3). Most efficient methods rely on the complementary hybridization of oligonucleotide capture probes to the targeted DNA sequences; these methods use capture arrays (31–33), or solution phase, also known as solution hybridization selection (3, 34, 35). Furthermore, the use of explorative probes, as provided by PhylOPDb, in sequence-capture methods allows the full identification and characterization of new taxa (3).

Despite its comprehensiveness, the probeset described in PhylOPDb, however, suffers from a lack of homogeneity that is the third important criterion of a probe design after sensitivity and specificity. Nevertheless, it has been demonstrated that *in silico* approaches for predicting the hybridization behaviour of microarray probes are limited: the only solution is to perform an extensive empirical testing of the probes (36). Moreover, *in silico* assessment of specificity is not sufficient and only an experimental validation can ensure a complete specificity. Prediction of cross-hybridizations strongly relies on both the database and the algorithm. Until now, no clear consensus has been obtained to classify 16S rRNA genes in a unique database. Furthermore, specificity tests suffer from the same complexity without a unique ‘universal’ tool. Moreover, for some experiments, low melting temperature probes could be used (37). Consequently, we preferred to provide a comprehensive probeset to let the users be able to select the best probes for their own experiments. Therefore, we recommend the PhylOPDb users to combine multiple tools (e.g. BLAST, SILVA-TestProbe, RDP-ProbeMatch) to confirm the specificity results of their selected probes.

Future work will be directed towards the development of specialized sets of phylogenetic oligonucleotides for specific ecosystems. Specificity tests are usually not performed against a suitable subset of sequences, primarily because of the lack of databases for microbial ecology. Depending on the environment studied, it would be more relevant to perform these tests against reduced databanks dedicated to specific ecosystems (e.g. soil, marine, freshwater and gut). Thus, additional probes specific to the targeted environments could be defined. Furthermore, specificity tests will be performed against reduced database limiting computational resources needs.

Probes provided through the PhylOPDb web interface were designed at the genus level but it would be interesting for some purposes such as PCR-based analyses to define probes at different phylogenetic levels. In addition, the current probe collection of the PhylOPDb will be extended to include 18S rRNA gene-targeted oligonucleotide for investigating fungal species within complex environments, where they may play crucial role.

## Funding

Auvergne Regional Council, the European Regional Development Fund, the French ‘Direction Générale de l’Armement’ (DGA) and the program Investissements d'avenir AMI 2011 VALTEX. Funding for open access charge: LIMOS, Clermont Université.

*Conflict of interest:* None declared.
